# IoT-Enabled Biosensors in Food Packaging: A Breakthrough in Food Safety for Monitoring Risks in Real Time

**DOI:** 10.3390/foods14081403

**Published:** 2025-04-18

**Authors:** Abdus Sobhan, Abul Hossain, Lin Wei, Kasiviswanathan Muthukumarappan, Maruf Ahmed

**Affiliations:** 1 College of Agriculture and Applied Sciences, Alcorn State University, Lorman, MS 39150, USA; 2 Faculty of Land and Food Systems, University of British Columbia, Vancouver, BC V6T 1Z4, Canada; abulh@mun.ca; 3 Department of Agricultural and Biosystems Engineering, South Dakota State University, Brookings, SD 57007, USA; lin.wei@sdstate.edu (L.W.); kas.muthukum@sdstate.edu (K.M.); 4 Department of Food Processing and Preservation, Hajee Mohammad Danesh Science and Technology University, Dinajpur 5200, Bangladesh; maruf@hstu.ac.bd

**Keywords:** IoT, food packaging, biosensor, food safety, digital data interface

## Abstract

The integration of biosensors and the Internet of Things (IoT) in food packaging is gaining significant interest in rapidly enhancing food safety and traceability worldwide. Currently, the IoT is one of the most intriguing topics in the digital and virtual world. Biosensors can be integrated into food packaging to monitor, sense, and identify early signs of food spoilage or freshness. When coupled with the IoT, these biosensors can contribute to data transmission via IoT networks, providing real-time insights into food storage and transportation conditions for stakeholders across each stage of the food supply chain, facilitating proactive decision-making practices. The technologies of combining biosensors with IoT could leverage artificial intelligence (AI) to enhance food safety, quality, and security in food industries, compared to conventional existing food inspection technologies, which are limited to assessing weight, volume, color, and physical appearance. This review focused on highlighting the latest and existing advancements, identifying the knowledge gaps in the applications of biosensors and the IoT, and exploring their opportunities to shape future food packaging, particularly in the context of 21st-century food safety. The review also aims to investigate the role of the IoT in creating smart food ecosystems and examines how data transmitted from biosensors to IoT systems can be stored in cloud-based platforms, in addition to addressing upcoming research challenges. Concerns of data privacy, security, and regulatory compliance in implementing the IoT and biosensors for food packaging are also addressed, along with potential solutions to overcome these barriers.

## 1. Introduction

Foodborne illnesses, especially food safety risks, pose significant concerns that threaten public health. According to the USDA, the annual cost spent only on foodborne illness mitigation was USD 17.6 billion in 2018, from USD 15.5 billion in 2013 [1]. These concerns have increased due to inefficiencies in the food supply chain, unsafe food processing and packaging, a lack of traceability, accelerating climate change, and rising consumer expectations of food production [2]. Additionally, the food industry is rapidly evolving worldwide, and the demand for packaged foods has simultaneously risen over time [3]. Therefore, there is an increasing demand for innovative technology with food packaging that can ensure monitoring of food safety both online and offline, early warning of emerging food risks, and mitigation of foodborne illness. In this perspective, one of the most promising concepts could be adopting advanced food packaging systems, which leverage biosensors and the IoT with food packaging to systematically monitor safety risks over time during food storage, transportation, and logistics [4].

The biosensor market, particularly electrochemical biosensors, has experienced significant growth. According to global market research, the electrochemical biosensor market showed an annual growth of 6.7% in 2023 compared to a growth of 3% in 2015 [5,6]. Biosensors in food safety fields have been effectively used to detect a wide variety of foodborne pathogens [7], food toxins and chemical contaminants [8], spoilage markers and food enzymes [9], and so on. These biosensors could be embedded directly into food packaging materials to provide continuous and real-time monitoring of the condition of the packaged food to indicate the presence or absence of food pathogens or contaminants by producing electrochemical and color-changing properties and conveying information on food quality deterioration. At the same time, the global IoT market was valued at USD 18.5 billion in 2022 and is estimated to grow to USD 27.7 billion by 2030, including an annual growth rate (CAGR) of 5.3% during this period [10]. The IoT can connect a wide range of digital devices and systems like computers and industrial appliances throughout a network [11]. By utilizing the IoT network, biosensors can allow for continuous data sharing so that the end users may rapidly address problems that are not expected to arrive. If food products are exposed to an abnormal temperature or humidity level during food transport or storage, the IoT system can immediately alert stakeholders, allowing them to take corrective action before food spoilage or contamination occurs [12]. Furthermore, the IoT and biosensors can enhance food traceability by providing detailed information about the origin and movement of food products, thereby helping to combat food counterfeiting and fraud [13].

Despite extensive research at the industrial and academic levels, research in IoT-enabled biosensors for food packaging is still in a very early developmental stage. Several challenges must be addressed before these technologies can be widely adopted and integrated into the food packaging industry. One of the primary concerns is data security and privacy [14]. As the biosensor and IoT systems incorporate data collection, transmission, and storage of temperature, humidity, and microbial content, they require robust data safety and security measures to prevent unauthorized access, hacking, or data manipulation. As the market for food packaging grows, there is a need for common protocols and interfaces that ensure different technologies to communicate and work together seamlessly through data security. It is necessary to look at the entire life cycle of biosensors and IoT-enabled food packaging, such as design and manufacturing, balancing cost, health, and environmental considerations.

This review aims to explore the advances, challenges, and opportunities of IoT-enabled biosensors in food packaging for 21st-century food safety. It provides an overview of the current state of food packaging technologies, focusing on integrating biosensors and IoT systems. The review discusses the various types of electrochemical biosensors that can be used in food packaging, their applications in monitoring food quality and safety, and the role of the IoT in facilitating real-time data transmission and decision-making. Furthermore, it emphasizes the technical, economic, and regulatory challenges that must be overcome to ensure the widespread adoption of smart food packaging technologies. Finally, the review highlights the potential opportunities for innovation in this field, including the development of fully autonomous packaging systems that can dynamically adjust to changes in environmental conditions, the creation of standardized platforms for biosensor and IoT integration, and the potential for greater sustainability in the food industry through improved supply chain management and waste reduction.

## 2. Biosensor Technologies and Opportunities

### 2.1. Biosensor Definition and Types

A biosensor is an analytical device consisting of a transducer and a receptor that can convert the input signals into a continuous output signal [15]. Receptors convert physical or chemical data into an energy form, while a transducer transforms this energy into a useful analytical signal, i.e., an electrical signal [16]. There are different kinds of biosensors used in applied science and engineering-related fields, but all of them might not be useful in application in food packaging sectors because of their unique properties, such as sensitivity, selectivity, and field of study. Some biosensors could be considered for food packaging to promote advanced food systems due to having target analyte detections, such as food pathogens, food spoilage biomarkers, food freshness levels, temperature, moisture, and so on. The biosensors that could be effectively used to detect and monitor various microbial contamination, packaged environmental conditions, and chemical changes to ensure the safety and integrity of food products are described in Table 1.

### 2.2. Biosensor Application for Food Packaging

#### 2.2.1. Pathogen Biosensors

A pathogen biosensor (PB) is an electrochemical biosensor developed depending on pathogen-targeted biological recognition elements, such as enzymes, antibodies, nucleic acids, and aptamers, among others, to detect food pathogens. These PBs are fabricated with electrochemical transducing materials, including graphene, carbon nanotube, MXenes, and conductive polymer [7]. The biological recognition elements and electrochemical transducing materials of PBs bind the food pathogen cells and subsequently produce an electrochemical signal, which can correlate the microbial loads and contamination levels [17,18]. PB has wide applications to detect a variety of foodborne pathogens, such as *E. coli*, *Salmonella*, and *Listeria*, among others. In food packaging, PB can play a crucial role in monitoring the food freshness, food integrity, and spoilage stages of packaged foods by detecting these foodborne pathogens [19]. For example, an immunosensor is a type of PB that uses antibody–antigen binding interactions to detect targeted foodborne pathogens [20]. The primary function of an immunosensor is to provide high antibody selectivity to the specific foodborne pathogen antigen cells and produce electrochemical signals as the output. There are different immunosensors that are useful for food packaging, such as ELISA biosensors, SPR-biosensors, optical immunosensors, and magnetic immunosensors [21,22,23,24]. In addition to foodborne pathogens, PBs can detect the presence of different analytes, such as allergens [25], spoilage indicators (i.e., histamines and volatile organic compounds—VOC) [26], mycotoxins [27], fats, and oils, and subsequently ensure food safety and quality. PB can be widely employed in food safety testing, enabling the rapid and accurate detection of harmful microorganisms such as *Salmonella*, *E. coli*, or *Listeria*. Their real-time detection capabilities can facilitate early contamination identification, reducing the risk of foodborne illnesses and improving consumer safety. Furthermore, in the field of food packaging, immunosensors can be integrated with food packaging film to continuously monitor chemical contaminants (e.g., Pb, As, and Cd) and have scope to ensure that only safe and high-quality food products reach consumers [28].

**Table 1:** Biosensor or sensor formats for food packaging.

Biosensor Type	Biosensor Format	Analytes	Sensitivity, Response	Remarks	Reference
Pathogen biosensor	Surface Plasmon Resonance (SPR) biosensor	*Salmonella typhimurium* *E. coli* O157:H7	10^3^ CFU/mL, 15 min 14 CFU/mL, 2 h	Low affinity with Salmonella and high affinity with *E. coli*	[17,29]
	Quartz Crystal Microbalance (QCM)	*Listeria monocytogenes*	336 nM, 60 min	Good sensitivity	[30]
	Impedance-based biosensors	*Campylobacter jejuni*	5 nM, 30 min	Very high sensitivity	[31]
	SWCNT biosensor	*Yersinia enterocolitica*	10^4^ CFU/mL, 30 min	Low affinity	[7]
	ZnO_NW/Au immunosensor	*Listeria monocytogenes*	8.3 × 10^2^ CFU/mL, 15 min	Low affinity and non-specific binding	[32]
	Pt@MnO_2_ nanowires	*Salmonella typhimurium*	13 CFU/mL, 1.5 h	High affinity and increased detection time	[33]
	rGO-TiO_2_-based biosensor	*Salmonella enterica*	10 CFU/mL, 5 min	High affinity with decreased detection time	[34]
	CuO_2_-Mxenes biosensor	*E. coli* O157:H7	30 CFU/mL, 50 min	High affinity	[35]
pH sensors	Ion-Sensitive Field Effect Transistor	pH in food samples	pH 3–10, 1 min	Sensitive to different pHs	[36]
	Optical Fiber pH Sensor	Food acids (e.g., citric, acetic acid)	105 CFU/mL, 1–5 min	Low affinity confirmed	[17]
	Electrochemical pH Sensor	General pH in liquids	±0.1 pH unit, <2 min	Very high sensitivity with rapid response	[37]
	Paper-based pH Sensor	Acids in beverages (e.g., juices)	pH 4–9, 1–5 min	Sensitive to different pHs	[38]
	pH-sensitive Fluorescent Sensor	Acids in canned fruits	pH 4–8, <1 min	Rapid response and sensitive to different pHs	[24]
	Enzyme-based pH Biosensor	Organic acids in dairy products	±0.05 pH unit, 2–5 min	Very high sensitivity	[39]
Gas Sensor	FET-type sensor	SO_2_	10 ppm, 3 min	High sensitivity	[40]
	Microcantilever sensor	H_2_S	1 ppm, 2 h	Very high sensitivity	[41]
	Pd-coated SnO_2_ nanofiber	H_2_	0.25 ppm, 40 s	Very high sensitivity with rapid response	[42]
	Carbon co-doped acetone sensor	Acetone	10 ppm, 100 s	High sensitivity with extended detection time	[43]
	Ethanol sensor	Ethanol	200 ppm	Good sensitivity	[44]
	Room temperature sensor	NH_3_	<1 ppm, 12 s	Very high sensitivity with rapid response	[45]
	Polypyrrole-based sensor	CO_2_	1.21 ppm, 72 s	High sensitivity	[46]
	2D-MoS_2_ FETs	NO_2_	2 ppb, 24 s	Very high sensitivity with rapid response	[47]
	ZnO/chemiresistive	H_2_S	2 ppb, 130 s	Very high sensitivity	[48]

#### 2.2.2. pH and Temperature Sensors

pH biosensors are used to monitor pH changes in foods and correlate them with food freshness [9]. They can also detect and quantify biological interactions compared to pH changes in foods to determine the food integrity (i.e., meat and fish integrity) for human consumption. There are different types of pH biosensors used to monitor the quality and safety of food products based on the pH change properties, as described in Table 1. Moreover, pH biosensors have a great opportunity to combine with fish, meat, and poultry packaging in measuring their product integrities by monitoring the pH levels of packaged products. The fundamental mechanisms of pH biosensors are these biosensors detect changes in hydrogen ion (H^+^) concentration based on the sensitive detected probe, which directly corresponds to pH levels and shows either electrochemical signals or color changes in response to the acidic or basic environment [49]. In addition, pH indicators use calorimetric dye to produce a color change in correlation with pH changes. While numerous studies have explored the concept of pH-indicating sensors [50], developing a pH-based indicator specifically for food packaging could be a promising area for future research because it is rapid, susceptible, specific, and reusable, making it valuable for ensuring food quality and safety.

A temperature sensor is a device that measures temperature changes and converts the data of temperature fluctuations into a readable output, typically in electrical, digital, color, or mechanical forms [47]. It has various applications, including food, agriculture, soil, industry, medicine, and environmental monitoring. Different kinds of temperature sensors have wide applications in food and agriculture (Table 1). Time–temperature indicators (TTI) are temperature sensors that monitor the storage conditions of temperature-sensitive products, such as meat and dairy, during transit [48]. Real-time data from these temperature sensors can alert supply chain managers to temperature deviations, preventing spoilage and maintaining product quality. Their application aligns with the trend toward food packaging, offering efficient monitoring solutions for perishable foods. It can detect temperature fluctuations during transportation and storage, helping companies take corrective actions before food quality degrades. Temperature sensors with eco-friendly materials can be a milestone in reducing electronic waste.

#### 2.2.3. Gas Sensors

A gas sensor is a device that detects and measures the concentration of food spoilage gases (NH_3_, H_2_S, CO_2_, O_2_, CO, H_2_, and CH_4_) in the surrounding environment [47,51,52]. It converts gas presence into an electrical signal that can be analyzed for various applications, including food safety, packaging, and quality control [53]. Gas sensors play a crucial role in detecting gas leaks within the packaging, helping to assess food quality. Additionally, these sensors provide a rapid and sensitive response for evaluating rancidity in meat products and detecting carbamate pesticides in harvested fruits and vegetables. The gas sensors are developed based on different mechanisms, such as electrochemical reactions, where target gases generate an electrical signal; metal oxide semiconductors, which change resistance upon gas exposure; and infrared absorption, where gases absorb light at specific wavelengths [41]. In food storage, gas sensors monitor gases such as oxygen (O_2_), carbon dioxide (CO_2_), ethylene (C_2_H_4_), and ammonia (NH_3_) to assess spoilage, ripeness, and packaging integrity [54]. By providing real-time data on gas concentrations, these sensors help maintain food safety, extend shelf life, and improve quality control in the food industry. Different types of gas sensors are mentioned in Table 1, and they have been widely studied to monitor ethanol, NH_3_, H_2_S, and acetone. In food packaging applications, these gas sensors can be constructed and renovated using miniaturized, flexible, and biodegradable materials to create eco-friendly and smart food packaging solutions. Different biodegradable nanomaterials, including graphene, carbon nanotubes, and lignin-based nanomaterials, can be sought for developing packaging sensors and improving sensitivity and selectivity to detect the spoilage gas, including NH_3_, H_2_S, and C_2_H_4_ [46,55,56]. These sensors may offer an advantage over any traditional detection methods, as they can be used in hazardous environments, exhibit high selectivity for specific gas molecules, and remain unaffected by electromagnetic interference. These innovations will revolutionize food safety, reduce waste, and enhance transparency in the supply chain.

## 3. IoT Technologies for Food Packaging Advancement

### 3.1. IoT and Internet Hub Application During Food Storage

**Figure 1:**
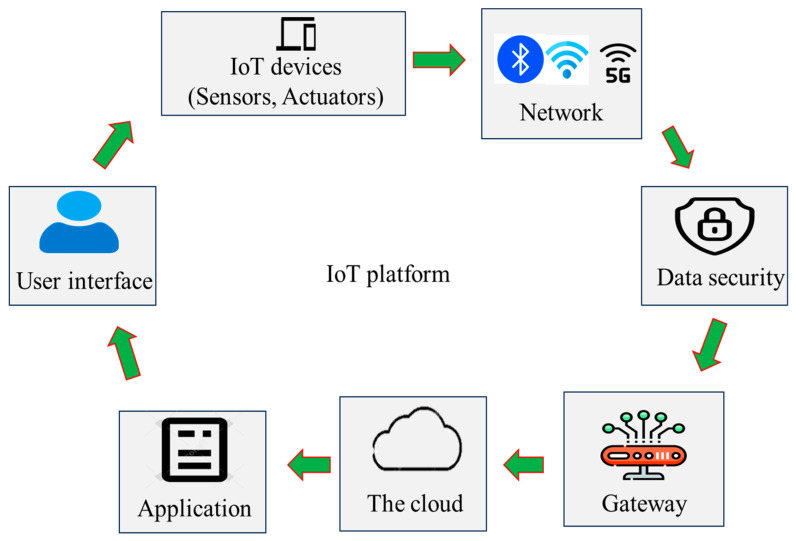
Interactions between the components of the Internet of Things [13].

The IoT is an internet system that connects computer devices and mechanical and digital machines for communication and data sharing [4]. The key components of the IoT are presented in Figure 1. Incorpoating the IoT into food packaging could be the pivotal step for building smart technologies. It can facilitate wireless communication, data sharing, and automation. IoT platforms have different applications to promote sustainable food safety practices and promotion [13]. For example, IoT-enabled devices, such as humidity and temperature trackers, as well as radio frequency identification (RFID) chips, may gather crucial data by monitoring microbial counts, humidity, water activity, temperature, and so on during food storage and help verify authenticity and monitor expiration dates, addressing challenges like counterfeiting and food waste [57]. With IoT-enabled packaging, food products can be traced throughout the supply chain in real time, providing transparency and accountability. For instance, QR codes or near-field communication (NFC) tags on packages can provide detailed product information, including origin, nutritional content, and preparation tips [58]. These data, shared throughout the IoT and Internet hubs, help producers, retailers, and consumers make informed decisions. During food recalls, IoT systems can quickly locate sources of contamination, reducing food risks and minimizing economic losses [59]. Another key advantage of the IoT in food systems is to track vital signs of food contaminants from remote areas, like homes and supermarkets, during warehouse food storage and send data to food stakeholders, allowing them to monitor food conditions continuously and make early decisions. This is particularly useful for managing food waste, where early detection of changes can prevent economic losses and allow for quicker intervention and better outcomes. IoT devices can be attached to packages so that their journey through a supply chain or distribution network can be traced, and their condition during this journey (e.g., their temperatures) can be monitored.

### 3.2. IoT for Food Transportation

**Figure 2:**
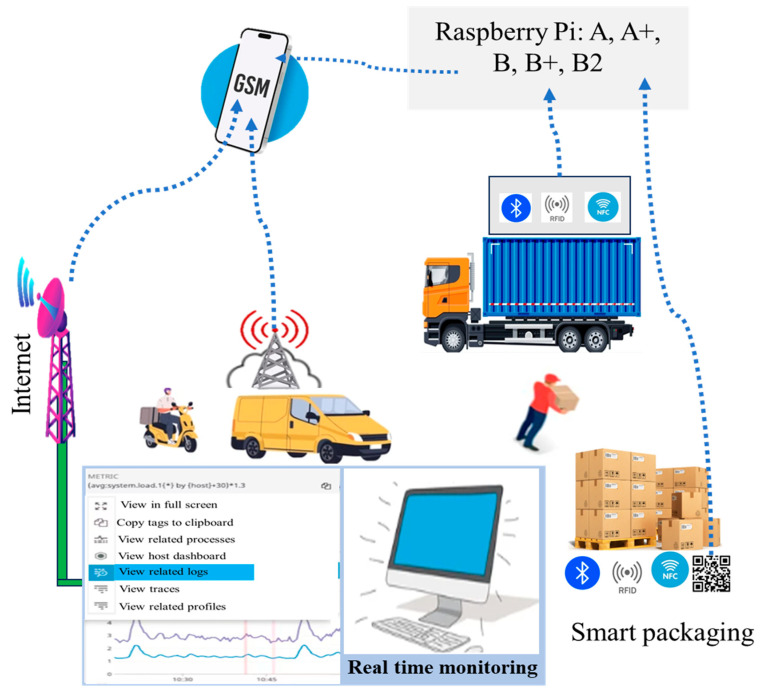
A schematic representation of food safety monitoring systems during transportation [65].

Around 30% of food products are wasted throughout the food supply chain [60]. As a result, millions of people experience hunger, even though such waste could be avoided. To tackle this food waste and reduce hunger, there is a pressing need for a developed system to monitor foodstuffs during transportation and storage. Transportation has a crucial role in the global food supply chain because of safe shipments of food products to processing facilities and, ultimately, to consumers. The need for reliable food transportation is increasing significantly because of the gradual expansion of international trade and online grocery delivery systems. For example, perishable food items like seafood, meat, dairy, and fresh produce require a temperature-controlled transportation system to ensure quality and safety. Improper temperature fluctuations during transit can lead to spoilage, causing financial losses and health risks [61]. The integration of the IoT and biosensors into food packaging transportation could detect and monitor food spoilage, contamination, and food freshness and can transport data on food quality to the stockholders to tackle the early steps of food spoilage [62]. A food safety monitoring system during food transportation is depicted on Figure 2. In addition, IoT-enabled biosensors in food packaging have the potential to monitor temperature, humidity, and pH during food transportation, and can track these parameters and send alerts if conditions deviate from set standards [63]. For example, IoT sensors will detect if the temperature during fresh fish transportation rises above a critical level, allowing timely corrective action. GPS-enabled devices can also provide real-time location tracking, ensuring timely delivery and reducing delays [64]. IoT-enabled biosensors in food packaging transportation will benefit the food industry and stakeholders by reducing waste and building consumer trust, thereby reducing food spoilage and transportation costs. Additionally, the transparency of food transportation monitored by IoT systems will be ensured, and confidence in the food supply chain will be improved from farm to fork.

### 3.3. Wi-Fi, Bluetooth, and 5G Data Transmission Technologies

The major data transmission technologies that potentially employ data sharing between devices, systems, and networks in smart healthcare, agriculture, and food applications are Wi-Fi, Bluetooth, 5G, and NFC [66]. Due to the data sharing efficacy, food packaging embedded with biosensors and the IoT could enable data (e.g., temperature, humidity, pathogens, and adulterants) to be transferred via Wi-Fi, 5G, and NFC to stakeholders in the food supply chain, including consumers, retailers, and other relevant parties.

**Figure 3:**
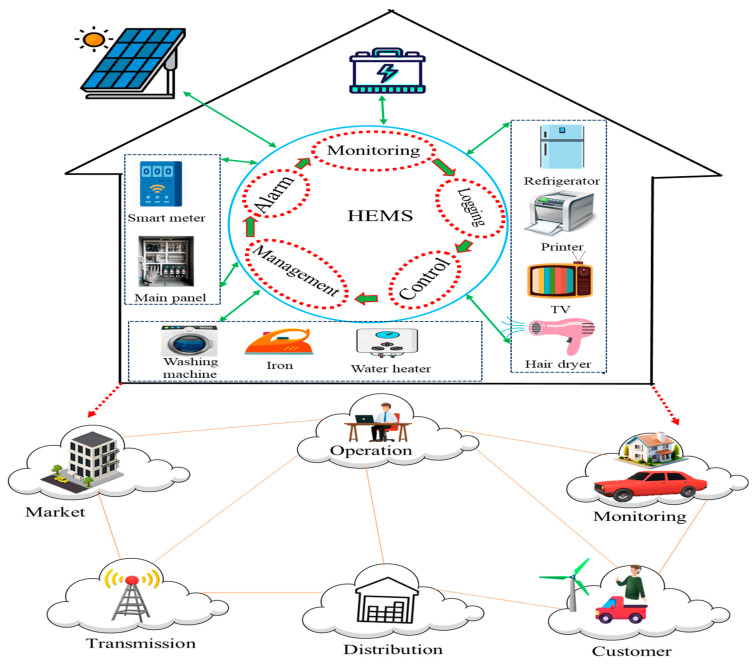
A schematic representation of a smart home system using HEMSs and smart grids [13].

Wi-Fi is a wireless networking technology that uses radio waves to provide high-speed internet (2.4 GHz and 5 GHz) and enable data exchange between devices [67]. They could distribute food storage data such as temperature and humidity to the food stockholder. Biosensor-equipped food with a connection to Wi-Fi networks can detect package storage conditions, humidity, and microbial contamination to share real-time data about the food’s freshness, prevent spoilage, and reduce waste [68]. Additionally, Wi-Fi helps in logistics by providing data on the location and condition of shipments, ensuring proper handling. Bluetooth is a short-range wireless technology for exchanging data between devices over a limited distance, typically up to 10 m [69]. It is energy-efficient and suitable for applications requiring low data transfer rates. On the other hand, 5G, the fifth generation of mobile network technology, offers faster data speeds, low latency, and massive device connectivity. It supports high-bandwidth applications and is ideal for the IoT. Bluetooth is useful in food packaging for enabling local communication between smart packages and nearby devices, such as smartphones or handheld readers. Home Energy Management Systems (HEMSs) and smart grids, integrated with Wi-Fi, 5G, and NFC technologies, can contribute to food safety opportunities by optimizing food storage conditions. A schematic representation of a smart home system using HEMSs and smart grids is presented in Figure 3. Wi-Fi and 5G enable HEMS to track temperature, humidity, and contamination risks in refrigerators and storage units. NFC allows consumers to scan food freshness, allergens, and expiration dates. Smart grids ensure a stable power supply, preventing food spoilage due to outages, while 5G ensures rapid updates on storage conditions.

### 3.4. Blockchain Food Traceability and Food Fraud

Blockchain is a distributed digital ledger that records transactions securely and transparently across a network of computers [4]. Unlike traditional databases, blockchain operates as a distributed system where data are stored in blocks that are linked together in a chain. Each block contains a list of transactions, a timestamp, and a unique cryptographic hash that ensures its authenticity and immutability [70]. Though this technology is widely used in various industries, including finance, healthcare, and supply chain management, due to its ability to enhance transparency and trust, it could potentially be used in food packaging industries. In the food industry, blockchain technology could enhance food traceability and prevent food fraud [71]. By integrating blockchain into the supply chain, stakeholders can track a product’s journey from farm to table in real time. In addition, consumers and stakeholders can scan QR codes linked to the blockchain and record data to verify product authenticity, including details about its origin, storage conditions, and certifications [72]. Additionally, blockchain can reduce fraudulent activities such as mislabeling, counterfeit products, and ingredient substitutions by providing an immutable record of transactions. This increased transparency not only ensures food safety but also strengthens consumer trust and regulatory compliance within the food industry.

The IoT and biosensors can be integrated with blockchain to rapid food traceability and prevent food fraud by providing real-time data on food quality, storage conditions, and supply chain movements [13]. In blockchain-based systems, biosensors embedded in food packaging or storage facilities monitor parameters such as temperature, humidity, pH levels, and contamination indicators [6]. The data collected from biosensors will be transmitted via the IoT to a blockchain-based system, ensuring stakeholders, from farmers to consumers, have access to accurate, tamper-proof information about a product’s journey. By scanning QR codes or RFID tags linked to blockchain records, consumers can verify the authenticity and safety of their food products. These combined initiatives to use blockchain, the IoT, and biosensors could be a milestone in future food packaging for real-time monitoring of packaged foods while also improving quality and safety. Additionally, blockchain’s decentralized nature prevents data manipulation, reducing fraudulent activities such as mislabeling, adulteration, and counterfeiting [73]. This synergy of IoT-enabled biosensors with blockchain technology may strengthen regulatory compliance and build consumer trust in the food supply chain. Although this concept of IoT-enabled biosensor application for food packaging is new, it has potential to benefit in large-scale food packaging in the future. Despite their synergic efforts providing benefits, they have some challenges, such as data privacy concerns, integration complexities, and scalability issues [14]. Additionally, it is a hurdle to increase the reliability of biosensors in diverse environmental conditions while also managing large amounts of data.

## 4. Challenges of IoT-Enabled Biosensors in Food Packaging

**Figure 4:**
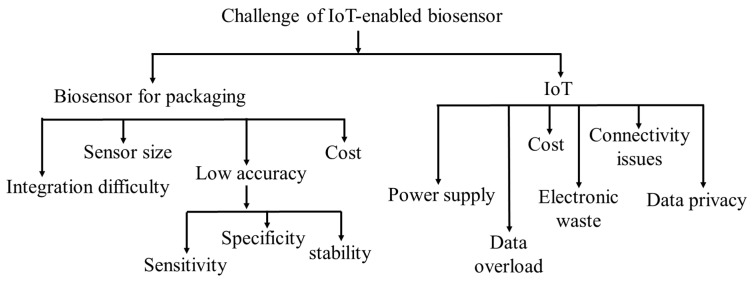
Challenges for IoT-enabled biosensors for developing food packaging technology.

IoT-enabled biosensors could have many potential scopes and opportunities to be employed in food packaging. These scopes and opportunities are critically very important to develop the food packaging system. However, some potential challenges and barriers have been found in the development of food packaging, depending on biosensors and the IoT. The most common challenges that have been described are as follows (Figure 4).

### 4.1. Power Management and Technological Barriers

Creating a unified information infrastructure for IoT-enabled biosensors in food packaging is significant. Biosensors need the power source to be operated for detecting and monitoring food pathogens, their surveillance, and packaged food conditions, as well as collecting data on packaging conditions and transmitting them through the IoT route for analysis, which involves a series of tasks. Furthermore, ensuring that the continuous power supply to the biosensor is properly connected with IoT networks to monitor food packaging conditions is necessary. However, providing a constant power supply to biosensors is challenging due to considering the cost, consumer acceptability, and convenience of packaged products. Traditional batteries available on the market may not be sufficient as they could deplete quickly or be bulky, impacting the cost of packaging. Therefore, alternative energy solutions, such as energy harvesting from environmental factors (e.g., light, motion, or temperature), are needed as the power supply source to overcome the limitation of power generation. However, exploring power from environmental sources (e.g., light, motion, or temperature) is still at the early developmental stage. Additionally, the development of low-power sensors and their designs, as well as efficient data transmission protocols, are necessary to minimize biosensor energy consumption. Striking a balance between maintaining biosensor accuracy and extending battery life is very important for the feasibility of IoT-enabled biosensors in food packaging without compromising the functionality or sustainability of the packaging itself.

### 4.2. Integration Barrier of IoT-Enabled Biosensor

IoT-enabled biosensors often face challenges in integrating with existing food packaging systems due to high costs and technical complexity. For example, integrating temperature-sensitive IoT biosensors into meat, fish, and milk cartons for real-time spoilage detection may require significant investment in sensor fabrication, compatibility testing, and system maintenance, calling for specialized materials, power sources, and advanced communication systems, which increases the overall cost of packaging [59]. It is suitable to develop a tiny biosensor for food production and packaging applications, to be integrated with food packaging depending upon the IoT. However, integrating these small biosensors into food packages is a major challenge because of their high sensitivity and specificity [51]. Nano-biosensors, such as miniature chips, are often invisible to the naked eye and can be chosen as packaging materials for monitoring food quality and packaged surrounding conditions. Nevertheless, despite their potential, nano-sensors have some limitations regarding their limited energy, which may not be appropriate for packaging field measurements. These limitations underscore the ongoing challenges in improving sensor size and developing effective integration techniques for biosensors in food packaging while also addressing the high production costs that cause difficulties in commercialization. These obstacles are associated with the manufacturing of IoT systems. Apart from these manufacturing barriers, several service issues must be considered, such as internet connectivity, interference, transmission losses, transmission range, network management, communication protocols, and lagging.

### 4.3. Data Security and Privacy Concerns

Integrating IoT-enabled biosensors into food packaging for food quality, safety, and freshness monitoring has significant data security and privacy challenges. As these technological systems of IoT-enabled biosensors are required to collect real-time data on food quality and safety as well as transmit them through IoT routes, they must often rely on wireless communication, which is vulnerable to hacking, data breaches, and unauthorized access [4]. As vast amounts of sensitive data are generated, transmitted, and stored through IoT systems, protecting this information is critical [14]. For instance, a food packaging system equipped with IoT-enabled biosensors monitoring the freshness of seafood might transmit sensitive data related to food freshness through unsecured networks, exposing it to cyberattacks [3]. Such breaches could lead to data manipulation, false alerts, or misuse of proprietary information. Ensuring end-to-end encryption, robust authentication protocols, and compliance with general data protection regulations (GDPR) is critical to address these challenges [74]. Another challenge is the scalability and cost-effectiveness of implementing IoT-enabled biosensors in food packaging [51]. While data security poses significant challenges, there are also opportunities for innovation in this area. Blockchain technology, for instance, can potentially enhance data security in IoT-enabled healthcare systems. By decentralizing data storage and creating tamper-proof records, blockchain can ensure that patient data remains secure and traceable, with only authorized individuals granted access [75]. Additionally, advances in AI-driven cybersecurity systems can help detect and respond to threats in real time, further protecting patient information from breaches [13].

## 5. IoT-Enabled Biosensor Opportunities

**Figure 5:**
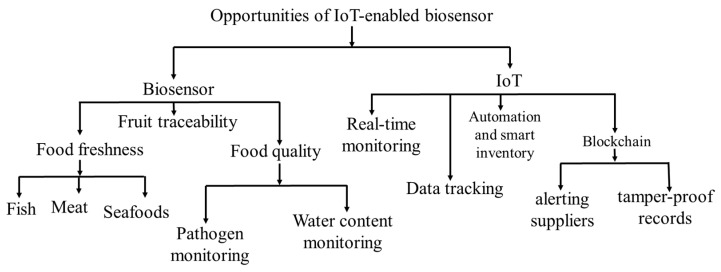
Opportunities for IoT-enabled biosensors in developing food packaging technology.

IoT-enabled biosensors have significant opportunities to rapidly detect food contaminants (such as toxins, foodborne pathogens, heavy metals, and so on), monitor food freshness, and provide signals to food authorities and stakeholders via cloud-based platforms. The most common opportunities that may be obtained from developing IoT-enabled biosensors for food packaging are shown in Figure 5.

### 5.1. Food Quality, Freshness, and Pathogen Investigation

Food quality is the attribute that is determined by evaluating the presence of foodborne pathogens, food nutritional values (proteins, fats, carbohydrates, vitamins, and minerals), taste, and textural appearance [73]. Food freshness is defined as how recently food was harvested, processed, or stored, affecting its edibility and shelf life [76]. Both attributes are essential for foods to ensure their taste, nutrition, and texture, reducing health risks and consumer satisfaction. Biosensors can detect harmful pathogens, toxins, and spoilage indicators such as pH changes, gas emissions, or microbial growth that are directly related to food quality and freshness, while the IoT facilitates wireless communication, data sharing, and automation [61]. These biosensors, coupled with IoT technology, may have opportunities to share data to a cloud platform, allowing for continuous monitoring of storage conditions, temperature, and humidity throughout the food supply chain and alerting stakeholders if food conditions deviate from safety standards. RFID tags can monitor temperature fluctuations and oxygen levels [74]. IoT equipped with these RFID have scope to be integrated into fish packaging to ensure fish freshness during transportation [77]. For instance, RFIDs can wirelessly measure food storage conditions (e.g., temperature and relative humidity) and share that data by utilizing a cloud platform of IoT regarding the status of stored foods. Thus, they can contribute to the pivotal role in managing food quality and safety.

Food integrity includes safety, quality, and authenticity [78]. Food safety applies to all those risks, chronic or acute, which can affect the consumer’s health. The authenticity of food means the food was not adulterated [79]. In the enclosed areas, fish and meat are spoiled by microorganisms, and the pH of these products is altered rapidly. IoT-enabled biosensors play a role in ensuring the integrity of meat and fish products throughout the supply chain. These sensors, integrated into packaging or storage systems, monitor key factors such as temperature, humidity, and gas composition to maintain the quality and safety of perishable foods. For example, biosensors can detect microbial contamination or pathogenic bacteria such as *Salmonella*, *E. coli*, or *Listeria* which can compromise the integrity of meat and fish [80].

Moreover, IoT-enabled biosensors in food packaging may provide considerable advantages over traditional packaging technologies, notably in terms of food quality, freshness, and disease detection. While existing packaging technologies, such as paper bags, plastic bottles, aluminum or tin cans, modified atmospheric packaging, etc., can provide some levels of protection against food degradation, they have notable limitations. For instance, they neither actively monitor food freshness nor detect food pathogens [51]. By embedding IoT-enabled biosensors into existing packaging, these challenges can be addressed through real-time monitoring of key factors (e.g., temperature, humidity, and pH levels) into existing packaging. Thus, it can provide instant alerts if the quality of packaged foods deviates from its optimal range. This packaging technology may fill the gaps in existing packaging by offering a data-driven approach to food safety, extending shelf life, and improving overall quality.

### 5.2. Food Allergen, Nutrition, and Dietary Recommendations

The IoT and biosensors can significantly detect food allergens and provide personalized nutrition and dietary recommendations [13]. Biosensors, designed to detect specific allergens (such as gluten, peanuts, dairy, or shellfish), can be combined with food packaging to trace allergens, typically using antibodies that bind to the allergens. The data from these biosensors could be transmitted via IoT networks to cloud-based platforms for real-time monitoring of allergens throughout the food supply chain. This ensures that potential allergens are detected quickly, reducing the risk of cross-contamination and accidental exposure [25]. IoT-enabled biosensors could be designed to interact with smartphones and provide real-time nutritional information, alerts, and personalized dietary recommendations. For example, consumers with lactose intolerance could receive a mobile notification about the presence of lactose in a dairy product before purchasing. This would improve the shopping experience and enable more informed decision-making. The ability to detect nutritional quality changes and loss in food products would also prevent malnutrition at retail and consumer levels, aligning with global sustainability goals. Adopting IoT-enabled biosensors in food packaging can significantly reduce operational costs by improving dietary supplements at multiple levels. These biosensors can automate quality assurance processes, reducing the need for labor-intensive inspections and manual testing. As production costs of IoT-based biosensors decrease, their integration into standard packaging will become economically viable for a broader range of food products.

Traditional labels, QR codes, and static nutritional information on the existing packaging can provide allergen and nutritional information [25], but they are limited to monitoring the presence of allergens in food. IoT-based biosensors embedded into existing packaging can overcome these limitations by providing real-time data on food allergen content, nutrient levels, and nutrient degradation, such as vitamin loss. In addition, they can convey personalized dietary recommendations based on an individual’s health data, ensuring consumers make informed choices.

### 5.3. Expiration Alert via Mobile Apps

One of the most promising opportunities for IoT-enabled biosensors could be monitoring microbial contaminations and chemical hazards as well as producing online or offline expiration signals [68]. The major food contaminations occur through food pathogens, e.g., *Salmonella*, *E. coli*, and *Listeria monocytogenes*, as well as harmful chemicals such as pesticides and antibiotics. IoT-based biosensors could rapidly detect these food contaminants and alert stakeholders via cloud-based platforms [81]. Integrating such biosensors with IoT technologies (e.g., mobile applications and Wi-Fi) might have potential opportunities to provide food safety updates to warehouse managers and consumers and reduce foodborne illness outbreaks [82].

Traditional expiration date labels on food products indicate the recommended date of food consumption but may not inform food spoilage stages or temperature fluctuations [51,79]. Therefore, these labels serve as a general reference rather than an absolute indicator of safety or quality. IoT-enabled biosensors have the potential to replace traditional expiration date labels by providing shelf life indicators based on actual food conditions. These biosensors can track parameters such as temperature, humidity, pH, and gas composition to determine the real-time freshness of perishable goods [3]. This would help consumers and retailers minimize food waste and optimize inventory management, ensuring food is consumed at its peak quality rather than discarded due to conservative best-before dates [4]. The IoT and biosensors in food packaging will enhance food traceability and transparency and monitor food transportation conditions [80]. If temperature, humidity, or other packaged food conditions deviate from optimal food storage conditions (e.g., temperature and humidity), IoT-based biosensors can trigger immediate corrective actions. Future innovations could involve blockchain-integrated biosensors that provide immutable food safety and quality records throughout the supply chain. This would facilitate better regulatory compliance, strengthen food authentication processes, and build consumer trust by providing verifiable information on food provenance.

### 5.4. Marketing Opportunities

The food packaging market is expanding gradually due to several factors, including rising consumer demand for processed foods, improved food safety, and the growing focus on food sustainability [51]. The packaged foods market was valued at around USD 350 billion in 2023 and is predicted to reach USD 500 billion by 2030, with a CAGR of approximately 5.5% [81]. It is estimated that about USD 34 billion will be spent on the IoT [13], while the biosensor market will grow and reach USD 50 billion in 2030 [82]. The IoT and biosensors have a great scope to significantly revolutionize the food packaging market by improving food safety and freshness monitoring. It is anticipated that the IoT will have grown faster than any other technologies by 2030 [83]. Although IoT-enabled food packaging market growth is tremendous for now and in the future, several obstacles must be addressed for effective market expansion. Firstly, the high cost of integrating the IoT and biosensors into food packaging materials can be a significant barrier for smaller companies or those in developing markets. This could limit the widespread adoption of food packaging. Secondly, developing eco-friendly packaging materials for integrated IoT-enabled biosensors that are both effective and cost-efficient remains a challenge. Addressing these obstacles will be crucial for realizing the full potential of the food packaging market.

## 6. Conclusions and Future Perspective

IoT-enabled biosensors in food packaging are a promising concept for offering food safety and quality for packaged foods. By combining biosensors with the connectivity of the IoT, these packaging technologies will be able to convey insights to stakeholders, from producers to consumers, regarding standards of food safety and transparency. Despite this promising progress, many challenges of IoT-enabled biosensor-enhanced food packaging remain for widespread adoption. One key challenge is the high cost of developing IoT connectivity with the biosensor, which needs to be controlled for the food packaging market. Research and development through academia and industry collaboration are required to overcome these challenges. Furthermore, addressing regional disparities in infrastructure and consumer awareness is needed to expand the reach of these technologies to underserved markets. As many engineering technologies and nano-molecules, e.g., blockchain, robot, and automation, have emerged and are being used along with sensors and actuators in the agricultural and food sectors, opportunities for such innovation can be used to impact the capabilities of IoT-enabled biosensors and create a way that not only ensures food safety but also engages consumers. By creating a multidisciplinary approach, IoT-enabled biosensors need to be safer, more efficient, and sustainable for the global food system.

The IoT-enabled biosensors could alternate traditional packaging systems to improve food safety, supply chain management, and consumer interaction. Progresses in nanotechnology, bioengineering, and material sciences are expected to produce more sensitive, accurate, and durable biosensors. Using emerging technologies, such as artificial intelligence (AI) and machine learning (ML), will improve data processing, allowing for better decisions on storage and distribution. Expanding the accessibility of IoT-enabled biosensors to underserved and small-scale producers is another critical priority. Developing affordable, easy-to-use systems for local needs will democratize the benefits of this technology, helping to ensure food safety and reduce losses in regions with limited resources. Collaboration across industries, governments, and academia will be vital in addressing global challenges such as food insecurity and climate change. By leveraging the synergistic potential of multidisciplinary research and cross-sector partnerships, IoT-enabled biosensors in smart food packaging have the potential to evolve into a cornerstone technology for achieving sustainable, resilient, and equitable food systems in the 21st century and beyond.

## Data Availability

No new data were created or analyzed in this study. Data sharing is not applicable to this article.
